# Stress Relief during Host Infection: The Phage Shock Protein Response Supports Bacterial Virulence in Various Ways

**DOI:** 10.1371/journal.ppat.1003388

**Published:** 2013-07-11

**Authors:** Andrew J. Darwin

**Affiliations:** Department of Microbiology, New York University School of Medicine, New York, New York, United States of America; University of North Carolina at Chapel Hill School of Medicine, United States of America

Inducible extracytoplasmic stress responses (ESRs) help to maintain the integrity and function of the bacterial cell envelope in unfavorable conditions. ESRs can also have highly specialized functions linked to virulence-associated systems directly. One of the most intriguing and yet enigmatic examples is the widely conserved phage shock protein (Psp) response [1]–[4]. This article outlines the significance of envelope stress and the roles of the Psp response in supporting bacterial virulence. This particular ESR might be critical for different reasons in different bacteria, with implications for both extracellular and intracellular pathogenesis, as well as processes that include antibiotic resistance and biofilm formation.

## Envelope Stress Responses Are Important for Bacterial Pathogens

Interaction with a mammalian host confronts bacteria with changes in temperature, osmolarity, and pH, which can cause cell envelope proteins to misfold and mislocalize, alter membrane properties, and even breach the membrane permeability barrier. The host can also attack the bacterial cell envelope with substances including bile salts, other surfactants, and antimicrobial peptides. To survive, bacteria can use their inducible ESRs to prevent lethal cell envelope defects. These responses have been studied most in Gram-negatives, where their importance during host interaction has been realized in recent years (reviewed in [5], [6]). The best-characterized ESRs are the widely conserved two-component system CpxAR and the RpoE extracytoplasmic function sigma factor system. These systems coordinate broad responses to aberrant cell envelope proteins [7]. However, both have also been linked specifically to virulence functions. For example, CpxAR regulates pili and type III secretion in pathogenic *E. coli*, type III secretion in *Shigella sonnei* [8]–[10], and type IV secretion in *Legionella pneumophila* [11]. The *Pseudomonas aeruginosa* RpoE system (known as AlgU/T) controls production of the exopolysaccharide alginate, a virulence factor in chronic lung infections [12].

Many bacteria remodel their envelope upon encountering the host. Often these changes involve the synthesis of complex envelope structures that are important virulence factors, such as T3SSs and pili. Improper assembly of these envelope structures might compromise the bacterial cell envelope and induce ESRs. One way to counter the potential envelope stress is for ESRs to downregulate these virulence factors, as proposed for the CpxAR systems of enteropathogenic *E. coli* and *Yersinia pseudotuberculosis* [13], [14]. However, another possibility is for an ESR to mitigate the stress while leaving virulence factor production unaffected. The Psp ESR does that in at least one of its roles to support bacterial virulence.

## The Phage Shock Protein (Psp) Response

**Figure 1:**
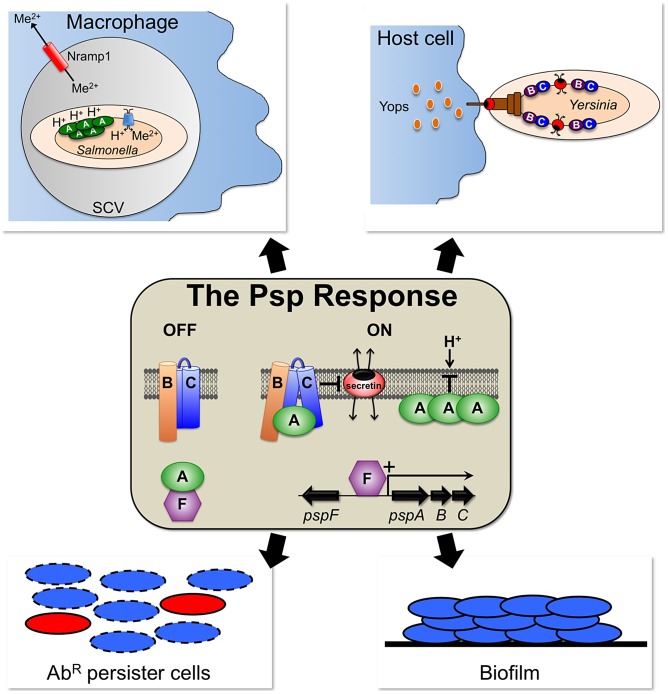
The Psp response and its involvement in various virulence-assocaied processes. In uninduced cells, PspA forms an inhibitory complex with the transcription factor PspF in the cytoplasm. An inducing trigger, such as the mislocalization of a pore-forming secretin protein, causes PspA to relocate to the cytoplasmic membrane, perhaps both in complex with PspBC and by making direct membrane contact. PspF induces *pspA* operon expression, leading to increased concentrations of PspA, -B, and -C that play roles in stress mitigation. PspA is believed to prevent proton (H^+^) leakage across the cytoplasmic membrane and maintain the proton motive force. This is thought to support *S*. Typhimurium virulence in Nramp1-positive mice by ensurng an energy supply for metal ion (Me^2+^) importers. PspB and -C prevent secretins from causing lethal cytoplasmic membrane permeability, which supports the T3SS-dependent virulence of *Y. enterocolitica*. The Psp response has also been shown to be required for the formation of biofilms and antibiotic-resistant (Ab^R^) persister cells in *E. coli*, although the Psp protein(s) involved has not yet been reported.

The “phage shock” name comes from the founding discovery that filamentous phage f1 infection massively induces the synthesis of an *E. coli* protein, which was named phage shock protein A (PspA) [15]. In *E. coli*, PspA is encoded by the *pspABCDE* operon, which is positively controlled by the transcription factor PspF (Figure 1). Filamentous phage infection induces *pspA* operon expression because a phage-encoded outer membrane pore-forming “secretin” protein (pIV) has a tendency to mislocalize within the *E. coli* envelope [3]. The Psp response is also induced by environmental shocks that can negatively affect the cell envelope. This, together with various other observations, has led to a hypothesis that the Psp system responds to, and helps mitigate, some aspect of cytoplasmic membrane perturbation [1]–[4].

Psp protein homologues, especially PspA, are present in Gram-negative and Gram-positive bacteria, as well as archaea and plants (reviewed in [16]). However, the Psp response has been studied most in the Gram-negative Enterobacteriaceae *E. coli*, *Yersinia enterocolitica*, and *Salmonella enterica* serovar Typhimurium (*S.* Typhimurium), where only PspF, -A, -B, and -C have been associated with robust phenotypes. One of their well-characterized functions is to autoregulate *psp* gene expression. This is achieved by PspA changing from an inhibitory interaction with PspF in the cytoplasm of uninduced cells, to a complex with the cytoplasmic membrane proteins PspB and -C of induced cells (Figure 1) [17], [18]. However, once *pspA* operon expression is induced, PspA, -B, and -C have additional roles in mitigating envelope stress. PspA has been focused upon due to its abundance, as well as experiments suggesting that it maintains the proton motive force (PMF) by preventing proton leakage across a damaged cytoplasmic membrane [19], [20].

## The Psp Response Supports Virulence by Preventing T3SS-Induced Envelope Stress in the Extracellular Pathogen *Yersinia enterocolitica*

The *Y. enterocolitica* Psp system is essential for virulence because of a specific connection to the Ysc-Yop T3SS. A *pspC* null mutant is avirulent in a mouse model of infection, and grows slower than the wild type in conditions that induce production of the Ysc-Yop system [21], [22]. This growth inhibition is caused by the outer membrane pore-forming component of the T3SS, YscC [22]. Like the phage pIV protein that induces the *E. coli* Psp system, YscC is a secretin. Indeed, when the *Y. enterocolitica* Psp system is intact, production of the native Ysc-Yop T3SS, or of only YscC, induces *pspA* operon expression [22]. Therefore, the Psp system is induced by production of the T3SS, and is then essential to mitigate a growth-inhibiting stress that its YscC secretin component can cause. The consequence is that a *psp* null mutant essentially kills itself during host infection by producing the Ysc-Yop T3SS to evade the immune response, but being unable to survive the stress that T3SS production causes.

Secretins have the unique ability to insert into either membrane of Gram-negative bacteria [23]. It is their mislocalization into the cytoplasmic membrane that induces *psp* gene expression and kills a *psp* null strain. Therefore, the toxicity of native T3SS production to a *psp* null strain suggests that endogenously produced YscC can mislocalize [22]. The cell death results from severe cytoplasmic membrane permeability, thought to be caused by the multimeric secretin channel (Figure 1) [24]. Remarkably, only the small membrane proteins PspB and PspC are required to prevent this toxicity from occurring, although it is not yet known how they do it [24]. Whatever the mechanism, the relationship between the Psp system and secretin mislocalization is highly specific. Secretin production induces *psp* gene expression without having much effect on any other genes in *Y. enterocolitica*, *E. coli*, or *S.* Typhimurium [25], [26]. It is surprising that PspA, the most abundant Psp protein, linked with maintenance of the PMF in *E. coli*, is not required to prevent secretin toxicity [24]. However, this does not mean that PspA is without an important role to play, or that it is always dispensable for virulence, as explained below.

## The Psp Response Supports Virulence by Maintaining the Proton Motive Force in the Intracellular Pathogen *S.* Typhimurium

Attention was drawn to the *S.* Typhimurium Psp system when it was found to be induced upon inactivation of RpoE [27]. Experiments suggested that PspA was compensating for the absence of the RpoE ESR by preventing a large drop in the PMF. The Psp system is also essential for *S.* Typhimurium virulence, but the explanation suggests a very different role from that in *Y. enterocolitica* [28]. *S.* Typhimurium is an intracellular pathogen that survives inside macrophages within a modified phagosome or *Salmonella*-containing vacuole (SCV; Figure 1). As a defense mechanism, the host uses the Nramp1 transporter to deplete phagosomes of divalent cations needed by bacteria. However, *S.* Typhimurium has energy-dependent metal ion importers to counter Nramp1. In Nramp1-positive mice an *S.* Typhimurium Δ*pspA* in-frame deletion mutant is severely attenuated [28]. The proposed explanation lies in the role of PspA in maintaining the PMF and so ensuring the energy to drive the metal ion importers. Consistent with this, in Nramp1-negative mice the Δ*pspA* mutation does not affect virulence [28]. Similarly, a Δ*pspA* in-frame deletion mutation has only a small effect on virulence of the extracellular pathogen *Y. enterocolitica* [22].

If PspA maintains the PMF of *S.* Typhimurium inside macrophages it could also be important for reasons beyond supplying energy to cation pumps. Lee and Groisman showed recently that the acidic pH inside the SCV drives increased ATP synthesis and elevates cytosolic ATP levels in *S.* Typhimurium [29]. The leader mRNA of the *mgtCBR* operon senses this elevated ATP concentration, inducing its expression and the production of proteins required for survival inside macrophages. If the absence of PspA decreases *S.* Typhimurium PMF inside macrophages it might also reduce PMF-driven ATP synthesis. This raises the intriguing but untested possibility that PspA might be important to ensure the induction of virulence genes such as *mgtCBR*.

## Other Links between the Psp Response and Virulence-Associated Processes

Observations suggest additional medically relevant roles for the Psp response (Figure 1). First, the *Shigella flexneri pspA* operon is highly induced during macrophage infection [30]. This suggests the possibility of a role for the Psp system in other intracellular pathogens, even though *S. flexneri* behaves differently from *S.* Typhimurium by escaping into the host cell cytoplasm. Second, the Psp system is important for biofilm formation by *E. coli* K-12 [31]. If this extends to pathogens there could be a connection between the Psp system and biofilm-mediated disease. Third, the Psp system has been linked to persisters in *E. coli* [32]. Persisters are dormant antibiotic-resistant cells implicated in chronic and recurrent infections. *E. coli* persisters can be induced by indole, a molecule produced upon entry into stationary phase. Indole also induces *pspA* operon expression, and a Δ*pspBC* mutant is defective for indole-induced persister formation [32]. The mechanism has not yet been investigated. Nevertheless, this exciting finding reveals a role for the Psp system in a phenomenon thought to have broad clinical significance.

It is becoming apparent that the Psp response has various fingers in various pies related to bacterial virulence. It seems likely that this extends beyond the examples highlighted here. Thus, the Psp system might represent an Achilles' heel for many pathogens. The challenge is to elucidate the molecular mechanisms underlying Psp protein functions and so understand how they affect the function and regulation of so many diverse virulence-associated phenomena.
